# Genetic Factors Contributing to the Pathogenesis of Essential Hypertension in Two African Populations

**DOI:** 10.3390/jpm14030323

**Published:** 2024-03-20

**Authors:** Kusha Kalideen, Brian Rayner, Raj Ramesar

**Affiliations:** 1 UCT MRC Genomic and Precision Medicine Research Unit, Division of Human Genetics, Institute of Infectious Diseases and Molecular Medicine, University of Cape Town, Cape Town 7704, South Africa; 2 Division of Nephrology and Hypertension, University of Cape Town, Cape Town 7700, South Africa; brian.rayner@uct.ac.za

**Keywords:** essential hypertension, South Africa, mixed ancestry, Xhosa, renin–angiotensin–aldosterone system, *AGTR1*, *AGT*, *ACE*, *CYP11B2*

## Abstract

The African continent has the highest prevalence of hypertension globally, with South Africa reporting the highest prevalence in Southern Africa. While the influence of genetic variability in the pathogenesis of hypertension is well described internationally, limited reports are available for African populations. This study aimed to assess the association of genetic variants and essential hypertension in a cohort of two ethnic South African population groups. Two hundred and seventy-seven hypertensive and one hundred and seventy-six normotensive individuals were genotyped for 78 variants. Genotyping was performed using the Illumina GoldenGate Assay and allele-specific polymerase chain reaction. The association of variants was assessed using the Fisher Exact test under the additive and allelic genetic models, while multivariate logistic regression was used to predict the development of hypertension. Five variants (*CYP11B2* rs179998, *AGT* rs5051 and rs699, *AGTR1* rs5186, and *ACE* rs4646994) were significantly associated with essential hypertension in the cohort under study. Furthermore, *AGTR1* rs5186 and *AGT* rs699 were identified as risk factors for the development of hypertension in both ethnic groups. In two ethnic South African populations, an association was observed between renin–angiotensin–aldosterone system (RAAS)-related genes and the development of hypertension.

## 1. Introduction

Essential hypertension (EH), defined as a systolic blood pressure (BP) of ≥140 mm Hg and/or diastolic BP of ≥90 mm Hg, is a major modifiable risk factor for cardiovascular disease and premature death worldwide [1,2,3]. Globally, the highest prevalence of EH is reported in the African region, with South Africa reporting the highest prevalence in Southern Africa [3,4]. As of 2016, the prevalence of EH in South Africa was 48.2%, an increase from 38.4%, as reported in 2012 [5,6]. A comparative analysis of two recent South African surveys (South African National Health and Nutrition Examination Survey (SANHANES) in 2012 and the 2016 South African Demographic and Health Survey (DHS)) reported a higher prevalence of EH in males, those of mixed ancestry, and those residing in urban areas [3].

The increasing prevalence of EH in South Africa has resulted in a rise in the number of patients being treated for EH. However, the proportion of patients achieving BP control has remained suboptimal, with an estimated 22.1% of treated patients achieving BP control in 2017 [7].

Several lifestyle factors have been attributed to the increasing prevalence and inadequate control of hypertension in African populations [3,4,7,8]. While these lifestyle factors may explain a portion of patients with inadequate control of BP, racial disparities in the clinical presentation and control of EH in African populations have been described [9,10,11,12]. A recent study concluded that African ancestry patients were more likely to develop EH at an earlier age and have a higher prevalence of EH, but less likely to have their BP controlled when compared to their Caucasian counterparts [10,12,13].

Severe and resistant hypertension has also been observed at greater levels in patients of African descent [10,11]. Often, these patients have biological differences due to a genetic predisposition to salt and water retention, supressed plasma renin activity, and differential response to anti-hypertensive drugs [11]. This genetic predisposition is hypothesised to be the result of historical environmental pressures and has been referred to as the Sodium Retention Hypothesis [14]. The Sodium Retention Hypothesis suggests that, evolutionarily, the capacity to retain salt provided a biological advantage and increased the fitness of salt retainers in tropical hunter-gatherer societies. However, with urbanisation and dietary changes in the modern era, the environmental pressure to retain salt was removed. As a result, the genetic predisposition to salt retention became disadvantageous and subsequently led to a rise in BP [11,14,15].

Despite the clinical differences observed in hypertensive patients of African descent, a limited number of studies have investigated genetic factors contributing to the pathogenesis of EH in these populations [16,17,18,19,20,21,22,23,24,25,26]. Genetic variants showing an association with EH in African populations are reported in Table 1.

To date, no genetic association with EH has been described in South African cohorts [18,35,36], though genetic associations in distinct South African ethnic groups have been described with blood pressure traits [37,38] and uncontrolled hypertension [16,39,40]. South Africa, with its rich ethnic diversity [41,42,43], presents a unique opportunity to unearth ethnic-specific associations, shedding light on the complex interplay between genetics and EH. With a focus on two South African ethnic groups, this study aims to investigate the association between well-described genetic variants, and the development of EH in Cape Town, South Africa.

## 2. Materials and Methods

### 2.1. Ethical Approval and Study Cohort

The University of Cape Town (UCT) Human Research Ethics Committee (HREC) approved this study (UCT HREC 328/2010). All individuals participating in the study provided informed consent. Individuals were recruited from Groote Schuur Hospital (GSH), an academic facility affiliated with the UCT in Cape Town, South Africa. Additional clinical information including the individuals’ age, sex, ethnicity, comorbidities (diabetes, a history of transient ischaemic attack, or cerebrovascular accident), and lifestyle factors (smoking status and alcohol consumption) was obtained.

Registered nurses drew two 5 mL Ethylenediaminetetraacetic acid (EDTA) tubes of blood by means of venesection from all individuals recruited into the study. Three consecutive BP measurements using a Dinamap (Soma Tech International, Bloomfield, NJ, USA) were taken and an average of the three readings were used as the final measurement of BP. Individuals were classified as hypertensive either based on the BP reading (the average systolic blood pressure reading was ≥140 mmHg and/or the average diastolic blood pressure was ≥90 mmHg), or because the individual recruited was a known hypertensive patient on treatment at the Hypertension Clinic at GSH. Normotensive individuals were classified as such when BP < 140/90 mmHg and the individual was not on any anti-hypertensive treatment at the time of study. As an exclusion criterion, no related patients were included in the cohort under study.

### 2.2. Identification of Genetic Variants under Study

Ninety-three (93) genetic single nucleotide polymorphisms (SNPs) postulated to influence the development of EH were identified via the literature and an analysis of the BP regulatory pathways. The complete list of variants identified may be found in the supplementary material (Supplementary Table S1).

### 2.3. DNA Isolation and Genotyping

A salting-out DNA isolation method [44] was used to extract genomic DNA from whole blood. The extracted DNA was quantified using the NanoDrop^®^ ND-1000 (Thermo Scientific, Wilmington, NC, USA). Two genotyping methods were utilized: the Illumina GoldenGate Assay (Illumina Inc, San Diego, CA, USA) and an allele-specific polymerase chain reaction.

#### 2.3.1. Illumina GoldenGate Assay

A total of 92 SNPs were genotyped using the Illumina GoldenGate Assay, a medium throughput genotyping method, as per the manufacturer’s protocol [45,46,47]. The analysis of the run was performed using the Illumina GenomeStudio Genotyping Module v2.0 software (Illumina Inc., San Diego, CA, USA), which uses a clustering algorithm for automated genotype clustering and calling [47,48]. Prior to genotype analysis, quality metrics were assessed for each SNP and each sample processed. Samples with low GenCall scores, indicative of a sample with poor performance on the assay, were excluded from the analysis. Furthermore, SNPS with low Cluster Separation Scores, low intensity for genotypes to be reliably called, and overlapping clusters were excluded from the analysis.

#### 2.3.2. Allele-Specific PCR

Intron 16 of the Angiotensin converting enzyme (*ACE*) gene harbours a 287 base pair polymorphism which could not be resolved using the Illumina Golden Gate Assay. To identify the presence or absence of this *ACE* sequence in this study, two allele-specific PCRs were sequentially performed, using a previously published protocol [49,50,51]. Further details on the protocol utilized are available in Supplementary Material S2.

### 2.4. Statistical Analysis

All statistical analysis was performed using R version 4.2.2 [52]. The Fisher exact method was used to test the association between the genetic variants under study and the hypertensive phenotype. For the association study, *p*-values of less than 0.00054 (post Bonferroni correction with 93 variants under study (*p* = 0.05/93)) were considered statistically significant.

Two genetic models were assessed; an additive genetic model (which assumes that there is an increased risk in disease per genotype) and an allelic genetic model (which assumes that one allele has a greater effect than the alternate allele) [53].

#### Multiple Logistic Regression

The generalized linear model (glm) function in R was used to perform multiple logistic regression to test the prediction of EH using the additive genetic model. Initially, all potential risk factors (clinical and lifestyle data, as described in Section 2.1) were included in the analysis. Backward elimination was used to remove each least significant predictor from the model. To assess model fit, both the predictive power of the model and the goodness of fit were used as indicators. The McFadden (pseudo) R2 was used to measure the predictive power of the model, while the goodness-of-fit indictors included the c-statistic and the Hosmer–Lemeshow statistic.

## 3. Results

### 3.1. Study Population

Four hundred and fifty-seven participants recruited into the study were genotyped. Four samples failed to meet the data quality metrics of the Illumina GoldenGate Assay and were thus excluded from the study. The resulting cohort under study included a total of 453 participants and classified as follows: based on the phenotypic criteria described in Section 2.1, 277 participants classified as hypertensive while 176 were classified as normotensive. The cohort could be further sub-classified based on self-reported ethnicity (Table 2). The sex distribution of the cohort is available in Supplementary Table S3a. The clinical and demographic data of the cohort are available in Supplementary Table S3b.

### 3.2. Genotyping Results

Seventy-nine variants were successfully genotyped. Nine variants failed to meet the recommended data quality metrics and therefore could not be confidently used in the analysis. An additional five variants could not be successfully genotyped for all patients in the cohort and were also excluded from the analysis.

Patients known with the *SCNN1B* R563Q (rs149868979 (ENaC)) variant were not included in this study. The cohort was assessed for this variant and all patients under study were genotyped as homozygous wild type. This mutation, previously identified by Rayner in a South African cohort [54], is associated with low-renin-low-aldosterone hypertension and pre-eclampsia in black African and mixed-ancestry individuals. Accordingly, 78 variants were used for statistical analysis.

### 3.3. Statistical Analysis: Association Study

The Fisher exact method was used to test associations between the 78 variants and EH under the additive genetic model. The results of these associations can be found in Table 3 and Table 4. With no stratification of the cohort, five variants (*CYP11B2* (rs1799998), *AGT* (rs5051), *AGTR1* (rs5186), *AGT* (rs699), and *ACE* (rs4646994)) were significant post Bonferroni correction (Table 3).

Fifty-two percent of the hypertensive cohort under study harboured the CC genotype in the *CYP11B2* rs1799998 variant, while fifty one percent of the normotensive cohort harboured the TT variant. The *CYP11B2* rs1799998 CC genotype was significantly associated with EH (*p* < 2.2 × 10^−16^) in this study (Table 3). This association remained when stratified by ethnicity (Table 4: Mixed Ancestry *p* = 1.045 × 10^−14^; Xhosa *p* = 1.042 × 10^−13^) and by sex (Supplementary Table S4a: Female *p* = 1.19 × 10^−15^; Supplementary Table S4b: Male *p* = 4.40 × 10^−9^).

Two variants within the *AGT* gene, rs5051 and rs699, were significantly associated with EH in the cohort (Table 3: *p* = 0.0001635 and *p* = 3.841 × 10^−5^, respectively). Both variants are known to be in linkage; however, the linkage disequilibrium blocks are reported to differ between Caucasian and Black populations [31]. Only the *AGT* rs699 variant demonstrated an association with EH in both the Mixed Ancestry (Table 4: *p* = 4.556 × 10^−5^) and Xhosa (Table 4: *p* = 4.775 × 10^−6^) ethnic populations. When the cohort was stratified by sex, the *AGT* rs699 variant was only associated with EH in females (Supplementary Table S4a: *p* = 5.79 × 10^−6^).

The *AGTR1* rs5186 variant and the *ACE* insertion/deletion polymorphism were significantly associated with EH in the unstratified cohort (Table 3: *AGTR1* rs5186 *p* < 2.2 × 10^−16^; *ACE* rs4646994 *p* = 4.323 × 10^−11^). The association was upheld when the cohort was stratified by ethnicity (Table 4: *AGTR1* rs5186 *p* < 2.2 × 10^−16^ in Mixed Ancestry and *p* = 1.831 × 10^−11^ in Xhosa; *ACE* rs4646994 Mixed Ancestry *p* = 9.446 × 10^−17^; Xhosa *p* = 1.395 × 10^−5^) and sex (Supplementary Table S4a for females: *AGTR1* rs5186 *p* < 2.2 × 10^−16^ *ACE* rs4646994 *p* = 1.00 × 10^−16^; Supplementary Table S4b for males: *AGTR1* rs5186 *p* < 2.2 × 10^−16^ and *ACE* rs4646994 *p* = 1.39 × 10^−15^, respectively).

#### 3.3.1. Allelic Genetic Model

The Fisher exact method was used to test associations of the major and minor alleles of each variant under study and EH. As was observed in the additive genetic model (Table 3), four variants were significant post Bonferroni correction: *CYP11B2* (rs1799998), *AGTR1* (rs5186), *AGT* (rs699), and *ACE* (rs4646994) (Table 5).

In this study, the C allele (rs1799998) of the *CYP11B2* gene was more prevalent in hypertensives than their normotensive counterparts, and conferred a 5.40 increased risk for the development of EH when compared to the T allele (Table 5: *p* < 2.2 × 10^−16^; 95% CI 4.010–7.324; OR 5.40). This effect was also significant when the cohort was stratified by ethnicity (Table 6: Mixed Ancestry *p* < 2.2 × 10^−16^; 95% CI 3.030–6.280; OR 4.35; and Table 6: Xhosa *p* < 2.2 × 10^−16^; 95% CI 5.140–16.071; OR 8.99) and sex (Supplementary Table S5a: Females *p* = 2.49 × 10^−6^; 95% CI 1.620–3.412; OR 2.35; Supplementary Table S5b Males *p* = 5.67 × 10^−16^; 95% CI 4.104–11.80; OR 6.89).

The *AGTR1* rs5186 A allele was associated with a decreased odds ratio for EH (Table 6: *p* < 2.2 × 10^−16^; 95% CI 0.090–0.178; OR 0.13) in the study cohort. This association remained when the cohort was stratified by ethnicity (Table 6: Mixed Ancestry *p* < 2.2 × 10^−16^; 95%CI 0.0747–0.173; OR 0.114; and Table 6: Xhosa *p* = 6.41 × 10^−12^; 95% CI 0.0859–0.288; OR 0.16).

The insertion allele in the *ACE* gene (rs4646994) was also found to confer a decreased risk for the development of EH (Table 5: *p* = 4.4 × 10^−6^; 95% CI 0.399–0.698; OR 0.529). On cohort stratification, this association was only significant in the Xhosa cohort (Table 6: *p* = 4.306 × 10^−5^; 95% CI 0.220–0.608; OR 0.367) and males (Supplementary Table S5b: *p* = 0.0001247; 95% CI 0.266–0.6683; OR 0.418).

#### 3.3.2. Multiple Logistic Regression

The model of best fit for the prediction of EH included four SNPs (*CYP11B2* (rs1799998), *AGT* (rs5051 and rs699), *AGTR1* (rs5186), and *ACE* rs4646994) (Table 7).

As per the fitted model, the *CYP11B2* r1799998 CT and rs1799998 TT genotype and *ACE* rs4646994 ID genotype resulted in a decreased risk of developing hypertension, with odds ratios of 0.2017, 0.0538, and 0.4329, respectively. These decreased risks were also observed in the allelic model (*CYP11B2* rs1799998 T allele and *ACE* rs4646994 I allele) (Table 5).

Conversely, the *AGT* rs5051 GT, *AGTR1* rs5186 AC and CC, and *AGT* rs699 CC genotypes resulted in an increased risk of developing hypertension, with odds ratios of 2.7688, 2.9494, 63.3178, and 10.6507, respectively. It is important, however, to caution the effects of the *AGTR1* rs5186 CC genotype and the *AGT* rs699 CC genotype due to large confidence intervals (AGTR1 rs5186 CC 95%CI: 23.7907–244.2167; and AGT rs699 95 CI 1.9382–72.7814). The model showed good discrimination (c-statistic: 0.91) and good fit (Hoslem–Lemeshow statistic: *p*-value = 0).

## 4. Discussion

This ground-breaking study delves into the intricate genetic landscape of EH within the diverse ethnic tapestry of South Africa. Recognizing the distinctive genetic makeup of the country’s population, this investigation focussed on two specific South African population groups: the Xhosa population group and the Mixed Ancestry population, an admixed population colloquially known as the Coloured population. This study was conducted in the Western Cape province, the third most populated province in South Africa. This region comprises 38.8% of Black South Africans (which includes the Xhosa ethnic group) and 41.2% Mixed Ancestry South Africans [55].

Our investigation identified significant associations between EH and five variants in genes related to the renin–angiotensin–aldosterone system (RAAS): *CYP11B2* rs179998, *AGT* rs5051, *AGT* rs699, *AGTR1* rs5186, and *ACE* rs4646994. These associations were evident under both additive and allelic genetic models, highlighting the robust genetic influence on EH within the South African context. Stratifying the cohort by ethnicity further unveiled nuanced associations. Notably, the *CYP11B2* rs179998, *AGT* rs699, *AGTR1* rs5186, and *ACE* rs4646994 variants demonstrated consistent associations with EH in both the Mixed Ancestry and Xhosa ethnic populations. Multinomial logistic regression pinpointed specific risk factors, emphasizing the intricate interplay between genetic variants and the development of EH in these distinct populations.

The RAAS system is a critical pathway in the regulation of BP and has been the focus of several studies investigating the genetic basis of EH [17,37,56,57]. This system is also the target of currently available hypertensive treatment [58]. The observed associations between RAAS variants and EH in our study support the Sodium Retention Hypothesis [59] and highlight the need for pharmacogenetic research of anti-hypertensive treatment in these population groups.

Delving into individual variants, the *CYP11B2* rs1799998 variant, located in the promoter region of the *CYP11B2* gene, exhibited associations with EH, thus corroborating studies linking this variant to higher plasma aldosterone-to-renin ratios and increased BP [60,61]. As an added layer to the complexity to the genetic landscape of EH, the prevalence of the C allele of the *CYP11B2* rs1799998 variant has demonstrated ethnic variation globally [30,62,63,64].

The *AGT* gene variants, rs5051 and rs699, showcased associations with EH, correlating to previously described studies. The rs5051 T allele is reported to correlate to higher plasma angiotensinogen levels in African populations, while the rs699 variant has been associated with elevated plasma angiotensin levels, contributing to increased BP [65,66]. The *AGTR1* rs5186 SNP in the angiotensin II receptor type 1 gene has also demonstrated ethnic-specific associations, aligning with the broader disparities observed in previous studies across Caucasian [67], Chinese [62], and African populations [17,29].

The *ACE* gene’s rs4646994 variant, an insertion/deletion polymorphism, unveiled opposing associations with EH, echoing the complex nature of genetic influences on BP regulation. This study highlighted disparities in associations across diverse ethnic cohorts previously reported [21,27,28,57,63,68,69,70,71,72,73], reinforcing the need for ethnicity-based considerations in genetic studies. The rs1799983 SNP in the *NOS3* gene presented sex-specific associations, emphasizing the importance of considering sex-specific genetic influences on EH described in a 2021 study on Brazilian women of African descent [74].

This study’s robust findings deviate from the existing literature on African populations [16,17,18], emphasizing the need for context-specific research. The distinct genetic associations uncovered herein could be attributed to the meticulous characterization of hypertensive patients attending a specialized clinic, shedding light on potential genetic predispositions within this subset. Additionally, this study marks the first exploration of genetic variants and hypertension in the Mixed Ancestry population, a significant contribution to the genetic understanding of EH in South Africa.

Acknowledging the study’s limitations, including a limited sample size for the Xhosa-speaking population group, we emphasize the necessity of expanding research to encompass a more extensive array of ethnic populations within South Africa. The identification of novel microRNAs in an African hypertensive population [66] underscores the importance of incorporating omics approaches in future studies to unveil previously undiscovered genetic variants contributing to EH.

In conclusion, our study reveals compelling associations between five genetic variants and EH in the Mixed Ancestry and Xhosa ethnic groups of South Africa. These findings underscore the importance of ethnicity in understanding the genetic underpinnings of hypertension. As we navigate the complexities of genetic influences on BP regulation, future research endeavours must adopt large-scale omics approaches in indigenous African populations, fostering a deeper understanding of the intricate genetic architecture governing EH in this unique demographic.

## Figures and Tables

**Table 1:** Genetic variants associated with EH in African populations.

Gene	Single Nucleotide Polymorphism (SNP)	Association	Reference
ACE	rs1799752 (also referred to as rs4646994)	DD genotype is involved in susceptibility to hypertension in Burkinabe and Ethiopian populations. D allele is associated with EH in Sub-Saharan African and Ethiopian populations.	[19,21,27,28]
AGTR1	rs5186	A allele is associated with EH in an Egyptian population.	[29]
ATP2B1	rs17249754	GG genotype had a higher risk of developing hypertension than AA+AG in Burkinabe.	[20]
CYP11B2	rs179998 (-344C/T)	T allele is associated with EH in Egyptian patients.	[30]
GSTM1 and GSTT1	(null)	*GSTM1*-null and *GSTT1*-null genotypes are potential factors to predict the development of EH in Egyptian patients.	[31]
GSTT1	(null)	*GSTT1*-null genotype is associated with EH in Burkinabe.	[32]
MTHFR	rs1801133 (C677T)	TT genotype is associated with the risk of hypertension in a Moroccan population. T allele associated with a predisposition to hypertension in a South-West Cameroonian population.	[22,23]
NOS3	rs2070744 -786T/C	CC genotype was associated with EH in a Sudanese population. C allele is associated with an increased risk of hypertension in an Algerian population, a Tunisian population, and a Sudanese population.	[24,25,33]
NOS3	rs1799983 G894T	TT genotype is associated with EH in a Moroccan population.	[34]

**Table 2:** Stratification of the study population.

Study Population	Mixed Ancestry	Xhosa	Total
Hypertensive Individuals	197	80	277
Normotensive Individuals	116	60	176
Total	313	140	453

**Table 3:** Variants significantly associated with EH in the study population.

Gene	Reference SNP Identification Number	Genotype	Hypertensive (N = 277)	Hypertensive %	Normotensive (N = 176)	Normotensive %	*p*-Value
*CYP11B2*	rs1799998 -344C>T	CC	145	52%	26	15%	<2.2 × 10^−16^
		CT	107	39%	60	34%	
		TT	25	9%	90	51%	
*AGT*	rs5051 -30-3273G>T	GG	24	9%	86	49%	0.0001635
		GT	134	48%	56	32%	
		TT	119	43%	34	19%	
*AGTR1*	rs5186 A1166C	AA	78	28%	124	70%	<2.2 × 10^−16^
		AC	56	20%	44	25%	
		CC	143	52%	8	5%	
*AGT*	rs699 T776C	TT	9	3%	96	55%	3.841 × 10^−5^
		TC	75	27%	56	32%	
		CC	193	70%	24	14%	
*ACE*	rs4646994 INDEL	II	50	18%	38	22%	4.323 × 10^−11^
		ID	121	44%	120	68%	
		DD	106	38%	18	10%	

**Table 4:** Variants significantly associated with EH in the Mixed Ancestry and Xhosa populations under study.

Gene	SNP ID	Genotype	Mixed Ancestry					Xhosa				
			Hypertensive (N = 189)	Hypertensive (%)	Normotensive (N = 116)	Normotensive (%)	*p*-Value	Hypertensive (N = 88)	Hypertensive (%)	Normotensive (N = 60)	Normotensive (%)	*p*-Value
*CYP11B2*	rs1799998	CC	89	47%	16	14%	1.045 × 10^−14^	56	64%	10	17%	1.042 × 10^−13^
	-344C>T	CT	79	42%	44	38%		28	32%	16	27%	
		TT	21	11%	56	48%		4	5%	34	57%	
*AGTR1*	rs5186	AA	42	22%	82	71%	<2.2 × 10^−16^	36	41%	42	70%	1.831 × 10^−11^
	A1166C	AC	50	26%	28	24%		6	7%	16	27%	
		CC	97	51%	6	5%		46	52%	2	3%	
*AGT*	rs699	TT	8	4%	24	21%	4.556 × 10^−5^	1	1%	0	0%	4.775 × 10^−6^
	T776C	TC	71	38%	36	31%		4	5%	20	33%	
		CC	110	58%	56	48%		83	94%	40	67%	
*ACE*	rs4646994	II	37	20%	22	19%	9.446 × 10^−7^	13	15%	16	27%	1.395 × 10^−5^
	INDEL	ID	90	48%	84	72%		31	35%	36	60%	
		DD	62	33%	10	9%		44	50%	8	13%	

**Table 5:** Alleles significantly associated with EH in the study population.

Gene	SNP ID	Allele	Hypertensive (N = 554)	Hypertensive (%)	Normotensive (N = 352)	Normotensive (%)	*p*-Value	95% CI	OR
*CYP11B2*	rs1799998 -344C>T	C	397	72%	112	32%	<2.2 × 10^−16^	4.010–7.324	5.40
		T	157	28%	240	68%			
*AGTR1*	rs5186 A1166C	A	212	38%	292	83%	<2.2 × 10^−16^	0.090–0.178	0.13
		C	342	62%	60	17%			
*AGT*	rs699 T776C	T	93	17%	104	30%	7.6 × 10^−6^	0.345–0.670	0.48
		C	461	83%	248	70%			
*ACE*	rs4646994 INDEL	I	221	40%	196	56%	4.4 × 10^−6^	0.399–0.698	0.529
		D	333	60%	156	44%			

**Table 6:** a. Alleles significantly associated with EH in the Mixed Ancestry population. b. Alleles significantly associated with EH in the Xhosa population.

a									
**Gene**	**SNP ID**	**Allele**	**Mixed Ancestry**						
			**Hypertensive** **(N = 378)**	**Hypertensive (%)**	**Normotensive** **(N =232)**	**Normotensive** **(%)**	***p*** **-Value**	**95% CI**	**OR**
*CYP11B2*	rs1799998 -344C>T	C	257	68%	76	33%	<2.2 × 10^−16^	3.030–6.280	4.35
		T	121	32%	156	67%			
AGTR1	rs5186 A1166C	A	134	35%	192	83%	<2.2 × 10^−16^	0.0747–0.173	0.114
		C	244	65%	40	17%			
b									
**Gene**	**SNP ID**	**Allele**	**Xhosa**						
			**Hypertensive** **(N = 176)**	**Hypertensive (%)**	**Normotensive (N = 120)**	**Normotensive (%)**	***p*** **-Value**	**95% CI**	**OR**
*CYP11B2*	rs1799998 -344C>T	C	140	80%	36	30.00%	<2.2 × 10^−16^	5.140–16.071	8.99
		T	36	20%	84	70.00%			
*AGTR1*	rs5186 A1166C	A	78	44%	100	83%	6.41 × 10^−12^	0.0859–0.288	0.16
		C	98	56%	20	17%			
*ACE*	rs4646994 INDEL	I	57	32%	68	57%	4.306 × 10^−5^	0.220 0 0.608	0.367
		D	119	68%	52	43%			

**Table 7:** Multinomial logistic regression results. * denotes *p*-values less than 0.05, ** denotes *p*-values less than 0.01, *** denotes *p*-values less than 0.001.

Gene	Coefficients	Estimate Std.	Error	z Value	Pr(>\z\)		OR	95% CI
	(Intercept)	−0.9927	0.9831	−1.010	0.31265		0.3706	0.04867–2.3612
	Gender Male	0.3958	0.3094	1.279	0.20081		1.4856	0.8133–2.7447
*CYP11B2*	rs1799998 CT	−1.6009	0.3730	−4.292	1.77 × 10^−5^	***	0.2017	0.0950–0.4119
	rs1799998 TT	−2.9223	0.4229	−6.910	4.83 × 10^−12^	***	0.0538	0.0226–0.1195
*AGT*	rs5051 GT	1.0184	0.4276	2.382	0.01722	*	2.7688	1.2181–6.5618
	rs5051 TT	−1.0739	0.7834	−1.371	0.17044		0.3417	0.0741–1.6306
*AGTR1*	rs5186 AC	1.0816	0.3541	3.055	0.00225	**	2.9494	1.4899–5.9991
	rs5186 CC	4.2242	0.5868	7.198	6.11 × 10^−13^	***	68.3178	23.7907–244.2167
*AGT*	rs699 TC	0.7752	0.8298	0.934	0.35015		2.1711	0.4545–12.1796
	rs699 CC	2.3656	0.9166	2.581	0.00985	**	10.6507	1.9382–72.7814
*ACE*	rs4646994 ID	−0.8372	0.3554	−2.355	0.01850	*	0.4329	0.2131–0.8627
	rs4646994 DD	0.4453	0.4546	0.980	0.32723		1.5610	0.6440–3.8519

## Data Availability

The data from the current article are available from the corresponding author upon reasonable request.

## Supplementary Materials

The following supporting information can be downloaded at: https://www.mdpi.com/article/10.3390/jpm14030323/s1, Supplementary material S1: Genetic variants under study; Supplementary material S2: ACE insertion-deletion methodology; Supplementary material S3: Sex stratification and baseline characteristics of the study cohort; Supplementary material S4: Variants significantly associated with EH when stratified by sex; Supplementary material S5: Alleles significantly associated with EH when stratified by sex.
