# Supplementary material for: Genetic Factors Contributing to the Pathogenesis of Essential Hypertension in Two African Populations
Source: J Pers Med. 2024 Mar 20;14(3):323. doi: 10.3390/jpm14030323 (PMC10971352; doi:10.3390/jpm14030323)
Supplement: Supplementary file 1 [file jpm-14-00323-s001.zip › jpm-2900250-supplementary.pdf]

Supplementary Material S1

Table S1. Genetic variants under study.

GENE	VARIANT	ALLELE	AA	GENE	VARIANT	ALLELE	AA	GENE	VARIANT	ALLELE	AA
ABO	rs495828	T>G	Upstream	***BDKRB2***	rs2227279	G1061A	Gly354Glu	***REN***	rs5707	A>C/G/T	Intron
ACE	rs2229839	C1052T	Pro351Leu	***CACNA1C***	rs16929470	C>T	Intron variant	***REN***	rs61746500	G22C	Pro8Ala
ACE	rs3730025	A765G	Tyr244Cys	***CACNA1C***	rs2239050	G>A	Intron variant	***REN***	rs6704321	C1207T(G)	Ala403Thr (Pro)
ACE	rs3730043	C1025T	Thr342Met	***CYP2D6***	rs5030656	CTT/-	Lys281Del	***SCNN1A***	rs13306616	G1382T	Pro461His
ACE	rs4298	C488T	Thr163Met	***CYP3A4***	rs2246709	G>A	Intron	***SCNN1A***	rs5742912	A1477G	Trp552Arg
ACE	rs4314	C1681T	Arg561Trp	***CYP3A4***	rs2740574	C>A/G/T	Upstream variant	***SCNN1A***	rs72657541	T806C	Met628Thr
ACE	rs4364	C2134A	Arg712Ser	***CYP3A5*3***	rs776746	T>C	intron	***SCNN1A***	rs72657550	G1736C	Gly579Ala
ACE	rs4976	T1331C	Ile444Thr	***CYP3A5*6***	rs10264272	G624A	Lys198=	***SCNN1B***	rs250563	C>T	Phe293Phe
ACE	rs4291	T>A	Upstream variant	***CYPIIB2***	rs1799998	G>A	Upstream	***SCNN1B***	rs41278184	C1136T	Thr379Met
ACE	rs4646992	Indel	Intron variant	***GNB3***	rs5443	C825T	Ser275Ser	***SCNN1B***	rs149868979	C1706G>A	Arg563Gln
ADD1	rs4961	G1378T	Gly460Trp	***Intergenic***	rs2820037	A>T	Intergenic	***SCNN1B***	rs72654326	C910T	Arg259Trp
ADRB2	rs1042714	G>C	Gln27Glu	***Intergenic***	rs11646213	A>T	Intergenic	***SCNN1B***	rs72654356	T2018A	Leu628Gln
AGT	rs11122576	A>T	Intron variant	***KLK1***	rs5518	T578A	Val193Glu	***SCNN1G***	rs5723	C19477G	Leu649Leu
AGT	rs4762	G620A	Thr207Met	***KNG1***	rs4686799	T>C	Intron	***SCNN1G***	rs5729		3'UTR
AGT	rs61751067	G1261A	Val421Met	***KNG1***	rs5030062	A>C	Non-coding transcript	***SCNN1G***	rs7200183		Intron
AGT	rs61751077	C1325T	Ser442Phe	***KNG1***	rs698078	A>G	Intron variant	***SCNN1G***	rs72646501	C776A	Thr259Asn
AGT	rs61762527	C1144G	Pro382Ala	***LDLR***	rs688	C1773T	Asn464Asn	***SCNN1G***	rs72647542	C1868T	Pro623Leu
AGT	rs61762537	C709T	Arg237Cys	***LOC124900725***	rs1458038	C>T	intron	***SLC12A3***	rs2399594	A>G	Intron
AGT	rs61762540	T251C	Leu84Pro	***LPL***	rs328	C1421G	Ser474NULL	***SLC14A2***	rs1123617	G>A	Val750Ile
AGT	rs61762541	G11C	Arg4Pro	***NOS1AP***	RS10494366	G>C/T	Intron	***SLC14A2***	rs3745009	G2638A	Ala880Thr
AGT	rs699	A8030G	Met268Thr	***NOS3***	rs1799983	T894G	Asp298Glu	***SLCO1B1***	rs4149056	T521A	Val174Glu
AGT	rs3889728	G>C	Intron	***NOS3***	rs2070744	C>T	Intron	***TGFB1***	rs1800471	G74C	Arg25Pro
AGTR1	rs12721225	G817T	Ala273Ser	***NOS3***	rs41508746	C1114T	Arg372Cys	***TNFRSF1A***	rs4149570	A>C	Upstream
AGTR1	rs13095608	T209G	Val70Gly	***NOS3***	rs61747096	C2831T	Ser944Leu	***TRPC7***	rs2277052	G>A	Intron
AGTR1	rs5182	C573T	Leu220Leu	***NPPA-AS1***	rs5065	A454G	NULL152Arg	***TRPC7***	rs2277052	G>A	Intron
AGTR1	rs5186	A>C	3' UTR	***NR2F2-AS1***	rs2398162	A>G	Non-coding transcript	***WNK1***	rs2107614	T>A	Intron
AGTR2	rs3729979	C812T	Pro271Leu	***NR3C2***	rs5523	A1331C	Asn444Thr	***WNK1***	rs2286007	C1994T	Thr665Ile
ATP2B1	rs17249754	C>T	Intron variant	***NR3C2***	rs5522	G538A	Val108Phe	***WNK1***	rs2277869		intron
BDKBR2	rs11847625	G>A/C	Intron	***PTGIS***	rs5629	G938T	Arg373Ag	***YEATS4***	rs315135	A>G	Intron
BDKRB2	rs1046248	C>T	Arg14Cys	***REN***	rs11571098	G>A	Arg33Trp	***YEATS4***	rs7297610		Intergenic
BDKRB2	rs1799722	C>T	5' UTR	***REN***	rs2368564	C>T	Intron	Intergenic	rs13278559	C>T	5' UTR

Supplementary Material S2

The ACE insertion/deletion was genotyped as previously described (49 – 51}. Briefly, all reactions were made up to a final volume of 25µl. A standard reaction contained 100ng of template DNA, 1.5 units (U) of GoTaq DNA polymerase (Promega, USA), 1 x GoTaq Polymerase Buffer (pH 8.5, 1.5mM $MgCl_2$ per reaction), a final concentration of 200 micromolar (µM) deoxyribonucleotide (dNTPs) (Bioline, USA), 5% Dimethyl sulfoxide (DMSO) (Merck, USA), 50mM KCl (Merck, USA), 10mM Tris-HCL (Merck, USA), 0.1% Triton X-100 (Merck, USA), a final concentration of 10pM of each primer (forward and reverse) and distilled water (Sabex) made up to the final volume.

Table S2a. *ACE* insertion/deletion primer pairs [49 - 51]

Primer Name	Primer Sequence
Forward Primer	5′ CTGGAGAGCCACTCCCATCCTTTCT 3′
Reverse Primer	5` GATGTGGCCATCACATTCGTCAGAT 3`
Forward Insertion Specific Primer	5` TGGGACCACAGCGCCCGCCACTAC 3`
Reverse Insertion Specific Primer	5` TCGCCAGCCCTCCCATGCCCATAA 3`

The standard cycling conditions used for the PCR included an initial denaturing step at 94° C for 5 minutes; 30 cycles of amplification, which involved a denaturing step at 94° C for 30 seconds; an annealing temperature of 67° C for 60 seconds; and an elongation step at 72°C for 2 minutes. A final step of 72°C for 5 minutes ensured the completion of the reaction. PCR products were detected using Agarose Gel Electrophoresis (AGE). The GeneRuler 100 base-pair (bp) molecular weight marker (Fermentas, USA) was employed for the resolution of fragments on an agarose gel. The marker was used at a concentration of 0.05 micrograms (µg)/µl. The PCR products were then analysed manually to determine the size of the product and genotyped accordingly. Insertion homozygotes presented with one band at 490 base pairs while insertion heterozygotes presented with one band at 490 base pairs and another at 190 base pairs. Deletion homozygotes only presented with a single band at 190 base pairs. The insertion-specific PCR yielded a product of 335bp in the presence of the insertion, while no product was observed in homozygous deletion samples.

Supplementary Material S3

Table S3a. Stratification of the study population by sex.

Study Population	Female	Male	Total
Hypertensive Individuals	163	114	277
Normotensive Individuals	114	62	176
Total	277	176	453

Table S3b. Baseline characteristics of the study population.

Study Population	Hypertensive	Normotensive
Average Age	36.39	36.87
Average BMI	30.16	31.9
Diabetes Status		
Diabetic	71	43
Not Diabetic	206	133
History of Cerebrovascular Accident or Transient Ischemic Attack		
Yes	32	18
No	245	158
Alcohol Consumption		
Current Drinker	144	101
No alcohol consumption	35	19
Past alcohol consumption	98	56
Smoking Status		
Current Smoker	125	74
Non-smoker	63	59
Past Smoker	89	43

Supplementary Material S4

Table S4a. Variants significantly associated with EH in the female cohort under study.

GENE	SNP ID	Genotype	Female				
			Hypertensive (N =163)	Hypertensive (%)	Normotensive (N = 114)	Normotensive (%)	p-Value
NOS3	**rs1799983** G894T	GG	11	7%	18	16%	3.84e-05
		GT	28	17%	40	35%	
		TT	120	74%	56	49%	
CYP11B2	**rs1799998** -344C>T	CC	89	55%	20	18%	1.19e-15
		CT	62	38%	42	37%	
		TT	12	7%	52	46%	
AGT	**rs5051** -30-3273G>T	GG	11	7%	50	44%	0.00011
		GT	72	44%	36	32%	
		TT	80	49%	28	25%	
AGTR1	**rs5186** A1166C	AA	54	33%	80	70%	< 2.2e-16
		AC	27	17%	28	25%	
		CC	82	50%	6	5%	
AGT	**rs699** T776C	TT	6	4%	18	16%	5.79e-06
		TC	39	24%	44	39%	
		CC	118	72%	52	45%	
ACE	**rs4646994** INDEL	II	33	20%	22	19%	1.00e-06
		ID	75	46%	82	72%	
		DD	55	34%	10	9%	

Table S4b. Variants significantly associated with EH in the male cohort under study.

GENE	SNP ID	Genotype	Male				
			Hypertensive (N =110)	Hypertensive (%)	Normotensive (N = 62)	Normotensive (%)	p-Value
CYP11B2	**rs1799998** -344C>T	CC	32	29%	6	10%	4.40e-09
		CT	45	41%	18	29%	
		TT	13	12%	38	61%	
AGT	**rs5051** -30-3273G>T	GG	23	21%	36	58%	4.72e-05
		GT	62	57%	20	32%	
		TT	13	12%	6	10%	
AGTR1	**rs5186** A1166C	AA	0	0.00%	44	71%	< 2.2e-16
		AC	29	26%	16	26%	
		CC	61	55%	2	3%	
ACE	**rs4646994** INDEL	II	13	12%	16	26%	1.39e-05
		ID	46	42%	38	61%	
		DD	51	46%	8	13%	

Supplementary Material S5

Table S4a. Alleles significantly associated with EH in the female cohort.

			**Female**						
**Gene**	**SNP ID**	**Allele**	**Hypertensive (N =326)**	**Hypertensive (%)**	**Normotensive N = 228)**	**Normotensive (%)**	**p-Value**	**95% CI**	**OR**
**CYP11B2**	**rs1799998**	C	201	65%	82	36%	2.49e-0.6	1.620 - 3.412%	2.35
	-344C>T	T	125	38%	146	64%			
**Intergenic**	**rs2820037**	A	243	75%	46	20%	4.24e-05	1.522 - 3.542%	2.30
	Intergenic	T	83	25%	182	80%			

Table S4b. Alleles significantly associated with EH in the male cohort.

			**Male**						
**Gene**	**SNP ID**	**Allele**	**Hypertensive (N =220)**	**Hypertensive (%)**	**Normotensive (N =124)**	**Normotensive (%)**	**p=Value**	**95% CI**	**OR**
**CYP11B2**	**rs1799998**	C	157	71%	30	24%	5.67e-16	4.104 - 11.80	6.89
	-344C>T	T	71	32%	94	76%			
**AGTR1**	**rs5186**	A	77	35,00%	104	84%	<2.2e-16	0.0536 - 0.1747	0.0987
	A1166C	C	143	65,00%	20	16%			
**ACE**	**rs4646994**	I	72	33%	70	56%	0.0001247	0.2600 - 0.6683	0.4180
	INDEL	D	148	67%	54	44%			
